# Identification and characterization of extrachromosomal circular DNA in large‐artery atherosclerotic stroke

**DOI:** 10.1111/jcmm.18210

**Published:** 2024-03-20

**Authors:** Kejie Chen, Yanqi Chi, Hang Cheng, Min Yang, Quandan Tan, Junli Hao, Yapeng Lin, Fengkai Mao, Song He, Jie Yang

**Affiliations:** ^1^ School of Public Health Chengdu Medical College Chengdu PR China; ^2^ Department of Neurology Clinical Medical College and The First Affiliated Hospital of Chengdu Medical College Chengdu PR China; ^3^ School of Bioscience and Technology Chengdu Medical College Chengdu PR China; ^4^ Department of Neurology, Sichuan Provincial People's Hospital, School of Medicine University of Electronic Science and Technology of China Chengdu PR China

**Keywords:** extrachromosomal circular DNA, ischemic stroke, large‐artery atherosclerosis

## Abstract

Extrachromosomal circular DNA (eccDNA) is a new biomarker and regulator of diseases. However, the role of eccDNAs in large‐artery atherosclerotic (LAA) stroke remains unclear. Through high‐throughput circle‐sequencing technique, the length distribution, genomic characteristic and motifs feature of plasma eccDNA from healthy controls (CON) and patients with LAA stroke were analysed. Then, the potential functions of the annotated eccDNAs were investigated using GO and KEGG pathway analyses. EccDNAs mapped to the reference genome showed SHN3 and BCL6 were LAA stroke unique transcription factors. The genes of differentially expressed eccDNAs between LAA stroke patients and CON were mainly involved in axon/dendrite/neuron projection development and maintenance of cellular structure via Wnt, Rap1 and MAPK pathways. Moreover, LAA stroke unique eccDNA genes played a role in regulation of coagulation and fibrinolysis, and there were five LAA stroke unique eccDNAs (Chr2:12724406‐12724784, Chr4:1867120‐186272046, Chr4:186271494‐186271696, Chr7:116560296‐116560685 and Chr11:57611780‐5761192). Additionally, POLR2C and AURKA carried by ecDNAs (eccDNA size >100 kb) of LAA stroke patients were significantly associated with development of LAA stroke. Our data firstly revealed the characteristics of eccDNA in LAA stroke and the functions of LAA stroke unique eccDNAs and eccDNA genes, suggesting eccDNA is a novel biomarker and mechanism of LAA stroke.

## 1 INTRODUCTION

Stroke is the second leading cause of death and disability worldwide, causing a huge burden to individuals and nations.^1^ Ischemic stroke (IS) accounts for approximately 80% of all stroke cases.^2^ Based on TOAST (Trial of Org 10172 in Acute Stroke Treatment) classification, the aetiologies include cardioembolic, large vessel atherosclerosis, small‐vessel occlusion, other determined causes and cryptogenic. Large‐artery atherosclerotic (LAA) stroke is mainly caused by atherosclerosis and thrombosis, which leads to narrowing or even occlusion of the lumen, causing IS. Currently, the accurate mechanism and effective management of LAA stroke is still challenging.^3^, ^4^ Therefore, there is a pressing need to identify effective biomarkers and therapeutic targets of LAA stroke.

Extrachromosomal circular DNA (eccDNA), a type of double‐stranded circular DNA, is derived and free from chromosomes. Since it was observed by Hotta and Bassel in 1965, eccDNA has been found in the blood and muscle of human,^5^ and the closed circular structure of eccDNA is highly stable and potent as biomarkers. It has been reported that eccDNA is involved in progresses of diseases, especially cancers, and could be potential biomarkers and therapeutic targets. Through segregating randomly in mitosis and replicating independently from mitosis of cells, eccDNA causes significant heterogeneity of tumour cells.^6^, ^7^ In patients with cancers, eccDNA may promote the expression of oncogenes and drug‐resistance genes of tumour cells,^8^, ^9^, ^10^ and lead to short survival.^8^

Various molecules/indexes have been identified as biomarkers of LAA stroke, including chemical biomarkers (e.g., low‐density lipoprotein cholesterol), inflammatory proteins (e.g., C‐reactive protein) and white blood cells (e.g., neutrophil‐to‐lymphocyte ratio).^11^ Recently, there are several researches reporting that the non‐coding RNAs in plasma are significantly involved in the development of stroke and associated with the risk of stroke.^12^, ^13^, ^14^ Compared with the non‐coding RNAs, eccDNAs have a longer half‐life, a more stable biological structure, and can carry complete genes and independently encode proteins,^15^ indicating eccDNAs are crucial molecules involving into the development of diseases. However, there is still lack of information on the role of eccDNA in non‐tumorous patients, like LAA stroke. Therefore, the present study was firstly conducted to investigate the characterizations of plasma eccDNA in the patients with LAA stroke, and identify the functional role of plasma eccDNA in LAA stroke. The data of the present study would provide important clues on the potentials of plasma eccDNA as biomarkers and therapeutic targets for LAA stroke.

## 2 PATIENTS AND METHODS

### 2.1 Study subjects

Five adult patients with LAA stroke were selected from First Affiliated Hospital of Chengdu Medical College between June 2021 and August 2022. LAA stroke was diagnosed according to the Guidelines for the Prevention of Stroke in Patients with Stroke and Transient Ischemic Attack. Five controls approximately age‐matched were enrolled from the health management centre of First Affiliated Hospital of Chengdu Medical College. Exclusion criteria were as follows: (i) more than 24 h after onset, and (ii) to have concomitant disease, including acute infection, immune diseases, cardiac disease and tumours. The study protocol conforms to the ethical guidelines of the 1975 Declaration of Helsinki and was approved by the Ethical Committee of First Affiliated Hospital of Chengdu Medical College. All study participants or their authorized representatives signed informed consent forms.

### 2.2 Purification and amplification of plasma eccDNA

A total of 10 mL of elbow vein blood from enrolled patients or control subjects were collected into EDTA anticoagulation tubes, centrifuged at room temperature, 4000 rpm for 10 min, and the supernatant was collected into EP tubes. Then, the dispensed supernatant was centrifuged again at 10,000 rpm for 10 min, and the supernatant was harvested as plasma in EP tube, labelled and stored at −80°C.

Cell‐free DNA (cfDNA) of plasma was extracted using QIAamp Circulating Nucleic Acid Kit (Qiagen) according to the instruction of manufacturer. For elimination of linear DNA and enrichment of eccDNA, plasma DNA was treated with exonuclease V (New England Biolabs) at 37°C for 30 min, and the remaining cfDNA was amplified using the REPLI‐g Single Cell Kit (Qiagen) at 30°C for 8 h. Then, the productions of eccDNA‐enriched DNAs were purified by column purification using MinElute Reaction Cleanup Kit (Qiagen) and quantified using Qubit 3.0.

### 2.3 Library preparation and sequencing

The eccDNA‐enriched DNAs were fragmented into 200–300 bp using sonication, and DNA libraries was constructed using Nextera XT DNA Library Preparation Kit (Illumina). Then, the libraries were subjected to sequencing on Illumina Novaseq 6000 using PE150.

Trimmomatic software were used to remove adapter and low quality reads from raw reads. The clean reads were then aligned to whole human genome sequence (CRCh38) using the BWA‐MEN program with default parameters.

### 2.4 Identification of eccDNA

The eccDNA was detected by the Circle‐Map, and filtering (split reads ≥ 1) was performed to improve the accuracy with parameters. All eccDNAs passing the filtering condition were used for further analysis.

### 2.5 Analysis of eccDNA

#### 2.5.1 General characteristics of eccDNA

After eccDNA molecules were located in the reference genome, their general characteristics, including the length distribution and the chromosome origin, were evaluated, and all plots were generated by the R package ggplot2. R package RIdeogram was used to visualize the chromosome origin of eccDNA. The repeated regions were downloaded from UCSC database, and reads in these region were count with deepTools, and finally, ratios of the reads were plotted using Graphpad Prism 9. The eccDNAs with size >100 kb were identified as ecDNAs.

#### 2.5.2 Genomic annotation of plasma eccDNA

Genes were annotated using Bedtools. LAA stroke unique genes/eccDNAs/transcription factors were identified as those represented in at least three LAA stroke patients but absent from the CON, CON unique genes/eccDNAs/transcription factors were defined as those represented in at least three CON but absent from the LAA stroke patients. The percentages of eccDNAs formed by genes (Top20) from LAA stroke patients or CON were visualized by Graphpad Prism 9. The chromosomal distribution of genes of differentially expressed eccDNAs were visualized by Graphpad Prism 9. Gene ontology (GO, http://www.geneontology.org/) and Kyoto Encyclopedia of Genes and Genomes (KEGG, http://kobas.cbi.pku.edu.cn/) pathway analyses were performed based on the genes associated with differentially expressed eccDNAs. The protein–protein interaction of ecDNA genes were analysed and visualized by STRING (www.string‐db.org/).

### 2.6 Motif signature

The 10 bp upstream and downstream sequences flanking the junction of all merged eccDNAs were analysed as a previously report.^16^ The start and end sequence motifs in R were generated using the R package ggseqlogo. The HOMER's findMotifsGenome.pl tool was used for Motif analysis. The input file is the peak file and the genome FASTA file. The DNA sequence is extracted according to the peak file, and the sequence is compared with the Motif database to obtain the Motif.

### 2.7 Statistical analysis

The eccDNAs with |log_2_ FoldChange| > 0 and *p* < 0.05 were identified as differentially expressed eccDNAs. Statistical analyses were conducted using R version ≥4.1.1. The difference between two groups was checked using Wilcoxon rank‐sum test; *p* value <0.05 was considered statistically significant.

## 3 RESULTS

### 3.1 Identification and distribution of eccDNA in plasma

Plasma eccDNAs from LAA stroke patients and CON were distributed across all chromosomes, and the number of plasma eccDNAs was positively associated with the density of genes distributing on all chromosomes (Figure S1A). Based on the number of eccDNAs per Mb on each chromosome, more eccDNAs were formed from chromosome 17 and 19 in comparison with others of both the LAA stroke patients and CON (Figure S1B). Among all chromosomes, higher coding genes per Mb were observed on chromosomes 17 and 19 (Figure S1C), consistent with the data of eccDNAs per Mb. Moreover, the differences of either eccDNAs per Mb or coding genes per Mb were not significant between LAA stroke patients and CON (*p* > 0.05).

### 3.2 Genomic characteristics of plasma eccDNA

The size distribution of eccDNAs from LAA stroke patients and CON showed similar patterns in Figure S2. Among all plasma eccDNAs identified in LAA stroke patients and CON, the size of approximate 99% eccDNAs was <1 kb, and the percentage of eccDNAs with size >100 kb was around 0.1% (Figure S2A). To investigate the difference of eccDNA size between LAA stroke patients and CON, length of eccDNAs from different size categories (<1 kb, 1–100 kb and >100 kb) was analysed. Across these size categories, there were not significant difference of eccDNA length between LAA stroke patients and CON (Figure S2B), and comparable size distributions were observed (Figure S2C–F) (*p* > 0.05).

**FIGURE 1:**
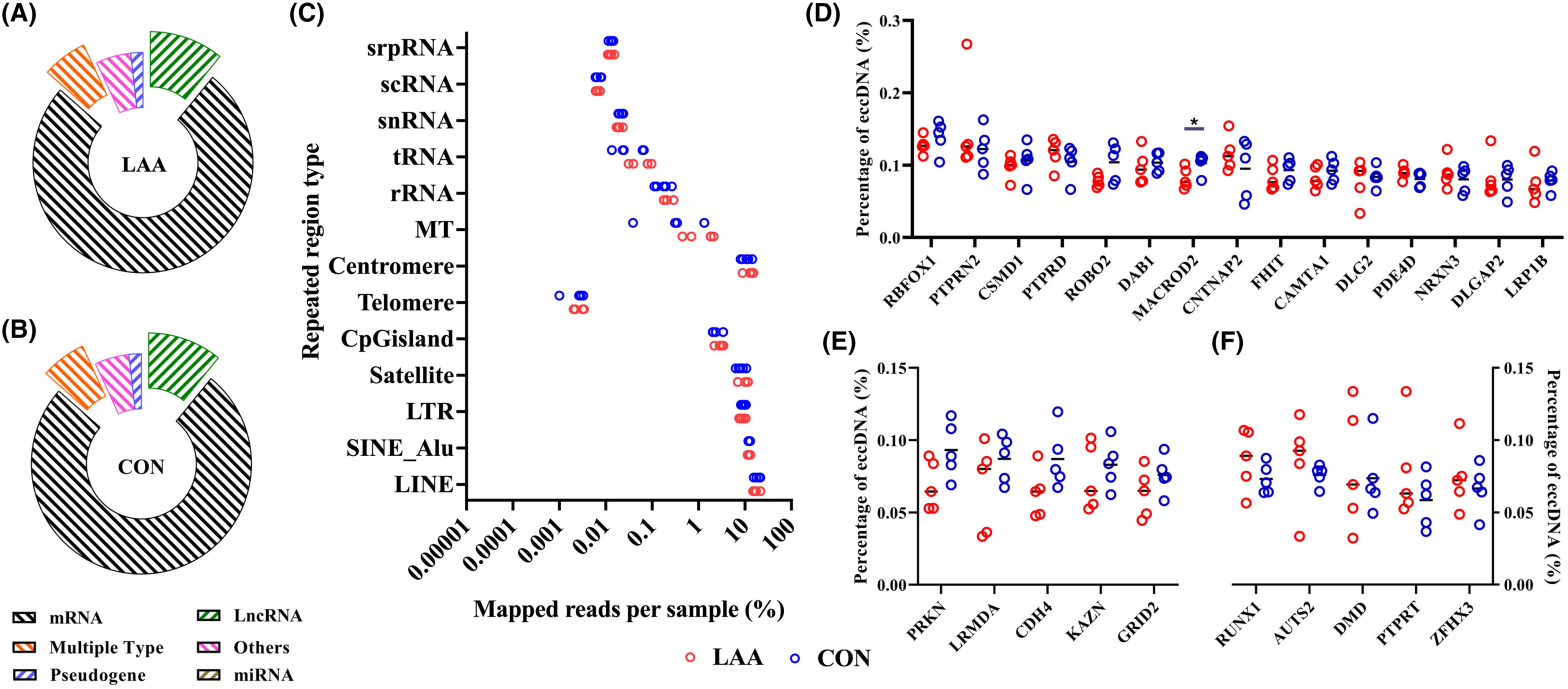
Genomic characteristics of annotated eccDNAs. Gene category of plasma eccDNA from the LAA stroke patients (A) and CON (B). (C) eccDNA annotation on repetitive regions. (D) The percentages of eccDNA from same genes (top 20) between the LAA stroke patients and CON. The percentages of eccDNA from unique genes (top 20) in the CON (E) or LAA stroke patients (F).

All eccDNAs identified in the present study were mapped to the reference genome to analyse their genomic characteristics (Figure 1). Of LAA stroke patients, most of plasma eccDNAs were formed from mRNAs, and least from miRNAs, similar with the CON (Figure 1A,B). A number of eccDNAs were mapped to various repetitive regions from human genome, including LINE, Centromere, SINE_Alu, LTR and Satellite and the distribution of repetitive regions of eccDNA between the LAA stroke patients and CON was not significantly different (Figure 1C) (*p* > 0.05).

Apart from that, the genes associated with eccDNAs were also explored. We analysed the frequencies of eccDNA genes and found that, of top 20 genes with highest percentages of eccDNAs, 75% genes were same between the LAA stroke patients and CON. Among these genes, the percentage of eccDNAs from MACROD2 in the LAA stroke patients was significantly lower than that in CON (*p* < 0.05) (Figure 1D–F).

### 3.3 Nucleotide motifs flanking eccDNA junctions

**FIGURE 2:**
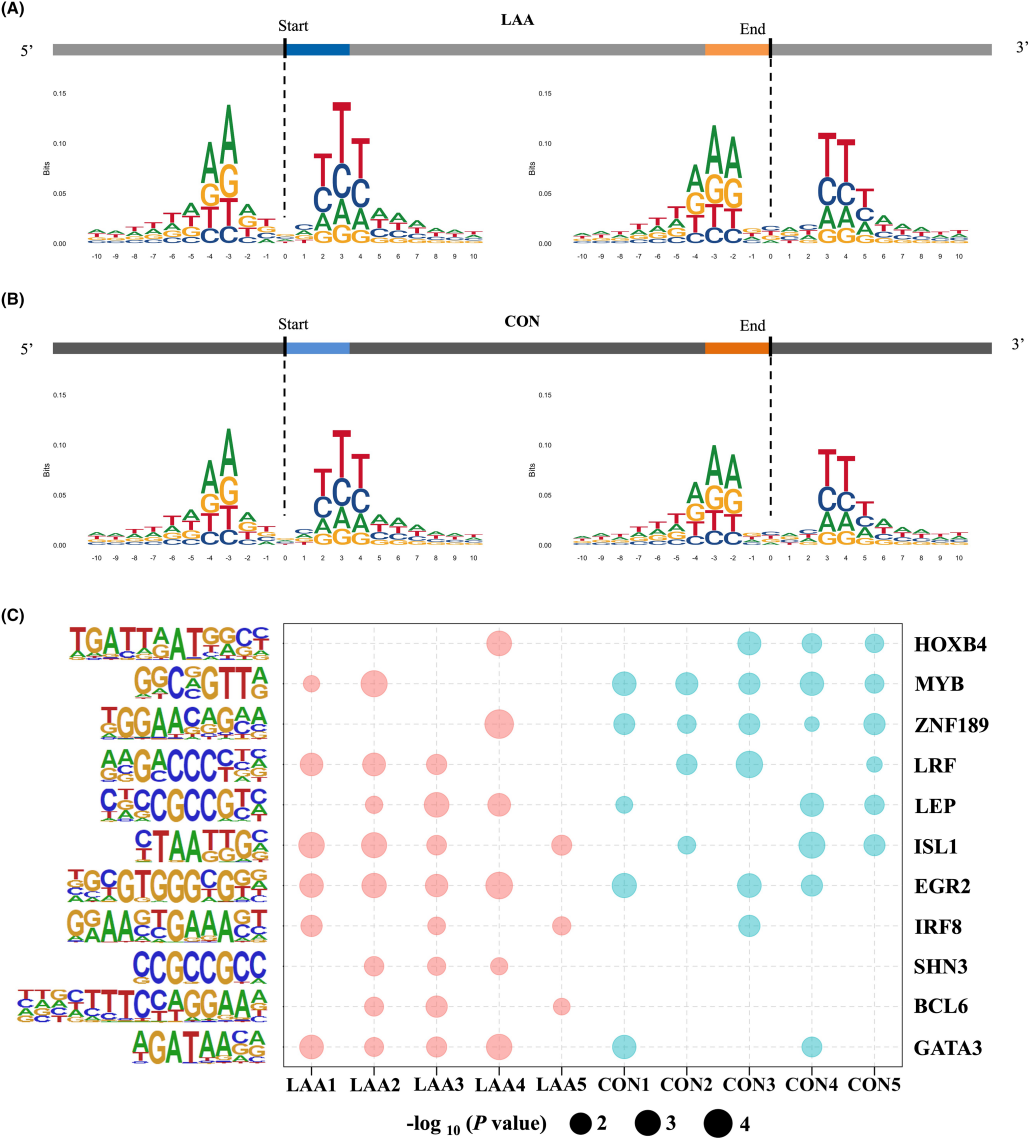
Motif analysis of eccDNA junctions. The nucleotide composition from 10 bp upstream to 10 bp downstream of the start and end position of the eccDNA junction in the LAA stroke patients (A) and CON (B). The frequency of base distribution at each position is plotted as a stacked histogram. The size of four bases at each position in the figure reflects their frequency. (C) Transcription factors matched in HOMER (*p* < 0.05) from 200 bp upstream to 200 bp downstream of the start and end position of the eccDNA junction in the LAA stroke patients and CON, respectively.

According to the stacked histogram, both the start and end positions of eccDNA junctions were flanked by a pair of high‐frequency trinucleotide segments with a low‐frequency interval (4 bp) in the LAA stroke patients and CON (Figure 2A,B). The position of interval was different between start and end of the LAA stroke patients and CON. The interval was from position −2 to 1 of start, while it was from position −1 to 2 of end. In addition, the frequencies of four bases of the trinucleotide segments showed similar compositions between the LAA stroke patients and CON (Figure 2A,B).

Thereafter, 200 bp upstream to 200 bp downstream of the start and end of eccDNA junctions from the LAA stroke patients and CON were used to analyse the function of motif via motif‐related transcription factors. There were two LAA stroke unique transcription factors, SHN3 and BCL6, which were represented in at least 3 LAA stroke patients but absent in CON (Figure 2C).

### 3.4 Functional analysis of differentially expressed eccDNAs

**FIGURE 3:**
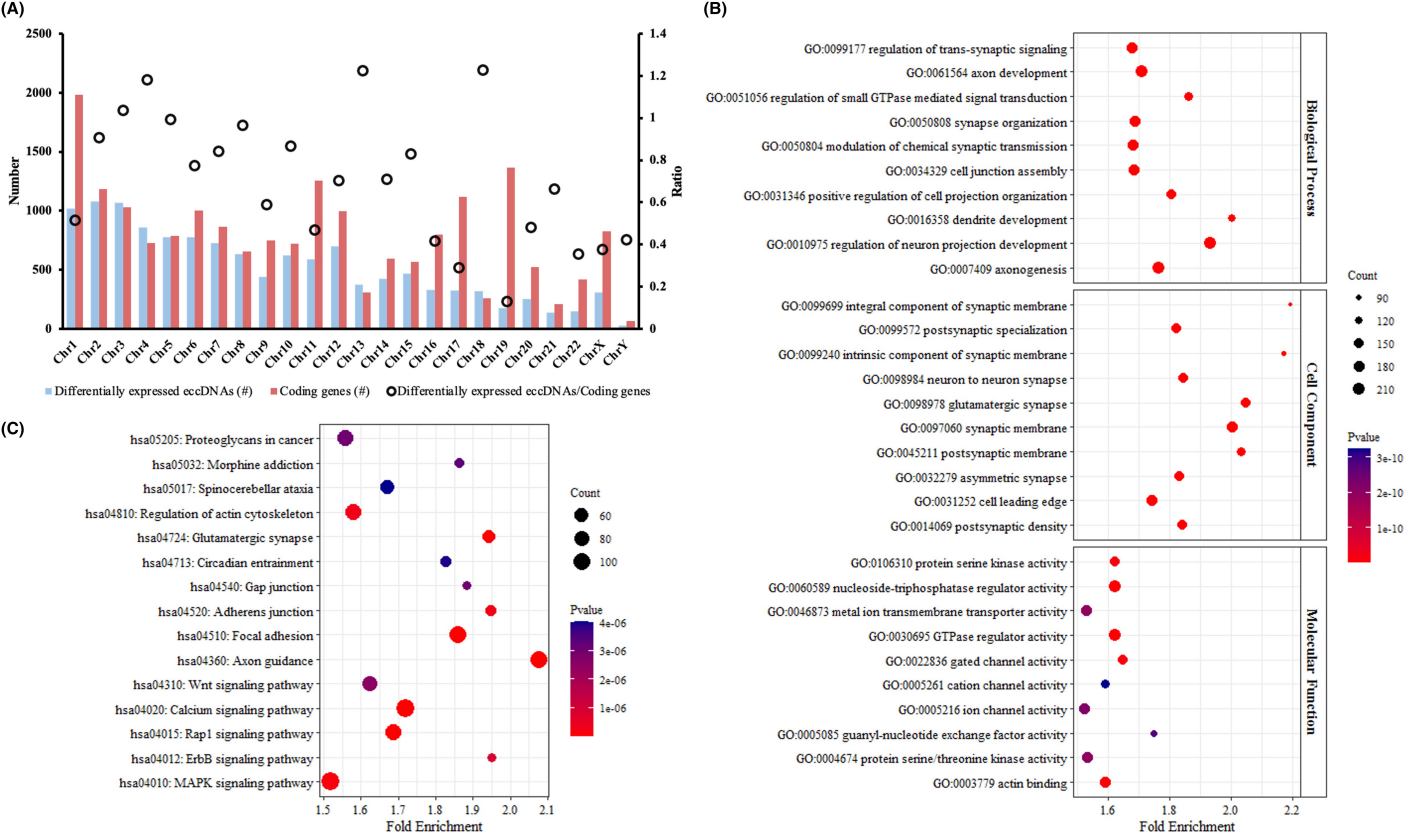
Chromosomal distribution (A), GO (B) and KEGG (C) analyses of genes related to differentially expressed eccDNAs between LAA stroke patients and CON. Top 10 terms with lowest *p* value were presented in GO annotation (*p* < 0.05), and top 15 pathways with lowest *p* value were visualized in KEGG pathway analysis (*p* < 0.05).

The genes related to the differentially expressed eccDNAs between LAA stroke patients and CON were unequally distributed on chromosomes (Figure 3A). According to the ratios of differentially expressed eccDNA number and coding gene number of each chromosome, the highest ratios were observed on chromosome 18, 13 and 4, while the lowest ratio was found on chromosome 19 (Figure 3A). Then, to investigate the function of eccDNA genes, the GO annotation and KEGG pathway analyses were performed for differentially represented eccDNAs between LAA stroke patients and CON. The GO annotation showed that the genes of differentially expressed eccDNAs between LAA stroke patients and CON were mainly from synapse membrane, and, through various kinases and channels, involved with neuronal function, including axon/dendrite/neuron projection development and synaptic signalling transmission (Figure 3B). Based on the KEGG pathway analyses, the pathways involved with genes of differentially expressed eccDNAs were significantly enriched in nervous system (axon guidance, glutamatergic synapse and spinocerebellar ataxia), maintenance of cellular structure (focal adhesion, adherens junction, gap junction and regulation of actin cytoskeleton), Wnt, Rap1, ErbB, MAPK and calcium signalling (Figure 3C).

### 3.5 Functional analysis of eccDNA unique genes

**FIGURE 4:**
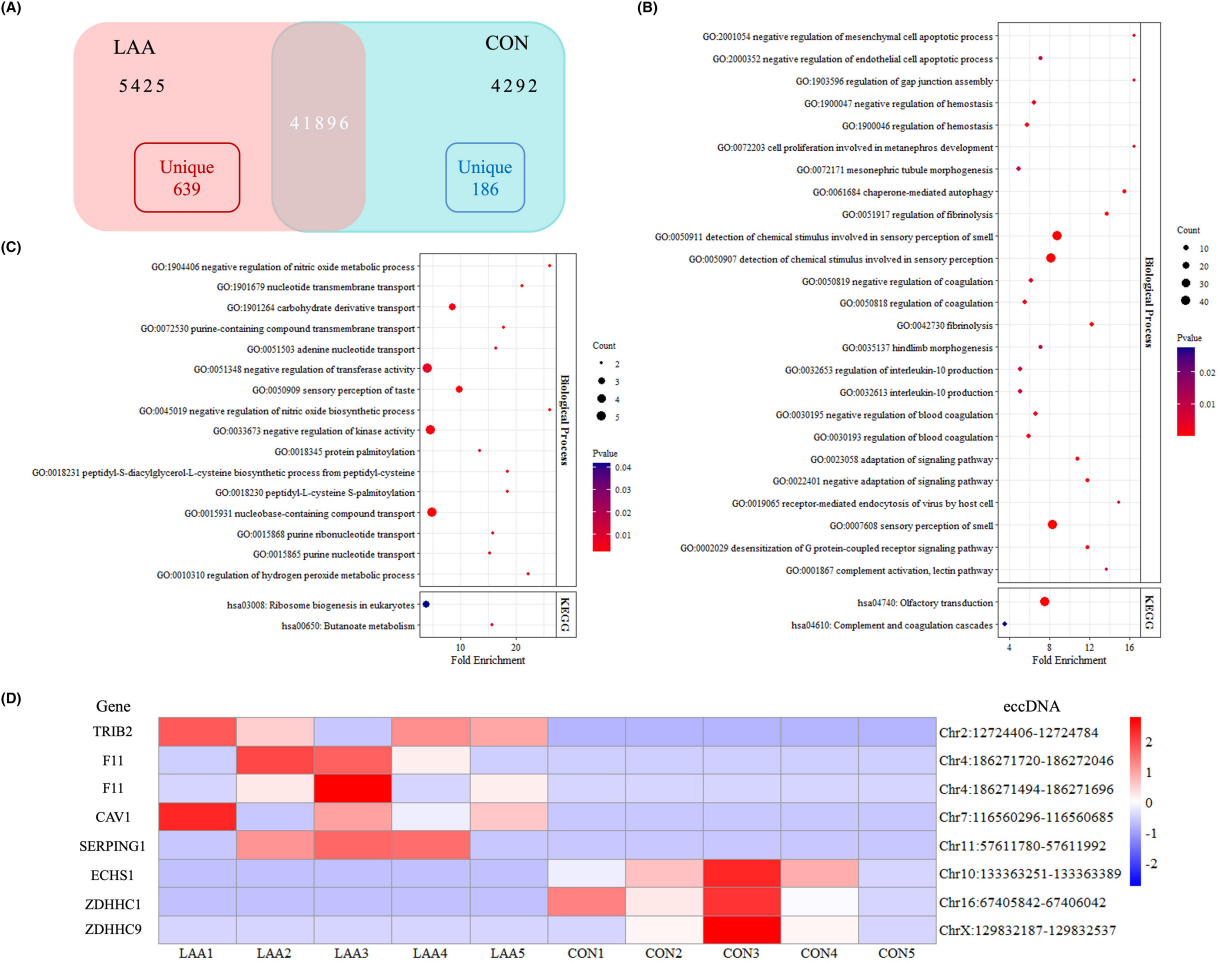
Number and functional enrichment of eccDNA genes of LAA stroke patients and CON. (A) Number of eccDNA genes and unique genes of LAA stroke patients and CON. (B, C) Biological process of GO annotation (*p* < 0.01) and KEGG pathway analysis (*p* < 0.05) of LAA stroke unique genes and CON unique genes, respectively. (D) Heatmap of eccDNAs derived from unique genes of LAA stroke patients and CON.

According to the gene annotation of all eccDNAs identified in the present study, a total of 51,613 genes were annotated in all study subjects. Of that, 41,896 (81.17%) genes were shared by the LAA stroke patients and CON, and 5425 (10.51%) and 4292 (8.32%) genes were only presented in plasma eccDNAs from the LAA stroke patients and CON, respectively (Figure 4A). To investigate the differentially expressed eccDNA genes in the LAA stroke patients, eccDNA genes that were represented in at least three LAA stroke patients but absent from the CON were defined as LAA stroke unique genes, CON unique genes were defined as those represented in at least three CON but absent from the LAA stroke patients. We found 639 LAA stroke unique genes and 186 CON unique genes in total (Figure 4A).

Through analyses of functional enrichment of unique genes, including biological process of GO annotation and KEGG pathway analysis, the role of either LAA stroke unique genes or CON unique genes could be explored. The data showed that the LAA stroke unique genes played a role in coagulation cascades and inflammation, including regulation of haemostasis/coagulation and fibrinolysis, complement activation and lectin pathway, and IL‐10 production (Figure 4B). In addition, the function of CON unique genes was mainly involved with regulation of nitric oxide and hydrogen peroxide, protein palmitoylation and butanoate metabolism (Figure 4C). The unique genes of eccDNAs enriched in each process were shown in Table 1.

**TABLE 1:** Unique genes of eccDNAs in the LAA stroke patients and CON.

Group	Term	Gene
LAA	Regulation of haemostasis/coagulation	CAV1/SERPING1/F11/PLAUR/VTN
	Regulation of fibrinolysis	SERPING1/F11/PLAUR/VTN
	Complement activation, lectin pathway	SERPING1/MFAP4
	Complement and coagulation cascades	SERPING1/F11/PLAUR/VTN
	Regulation of IL‐10 production	BCL3/LGALS9/PRG2/TRIB2
CON	Regulation of nitric oxide	MIR199A1/ACP5
	Regulation of hydrogen peroxide	ZNF205/RAC2
	Protein palmitoylation	ZDHHC1/ZDHHC9
	Butanoate metabolism	ECHS1/ACSM5

Based on the functional enrichment, the eccDNAs originated from unique genes of LAA stroke patients and CON were also studied to explore the potential biomarkers. There were five LAA stroke unique eccDNAs (Chr2:12724406‐12724784, Chr4:1867120‐186272046, Chr4:186271494‐186271696, Chr7:116560296‐116560685 and Chr11:57611780‐5761192) and three CON unique eccDNAs (Chr10:133363251‐133363389, Chr16:67405842‐67406042 and ChrX:129832187‐129832537) (Figure 4D).

**FIGURE 5:**
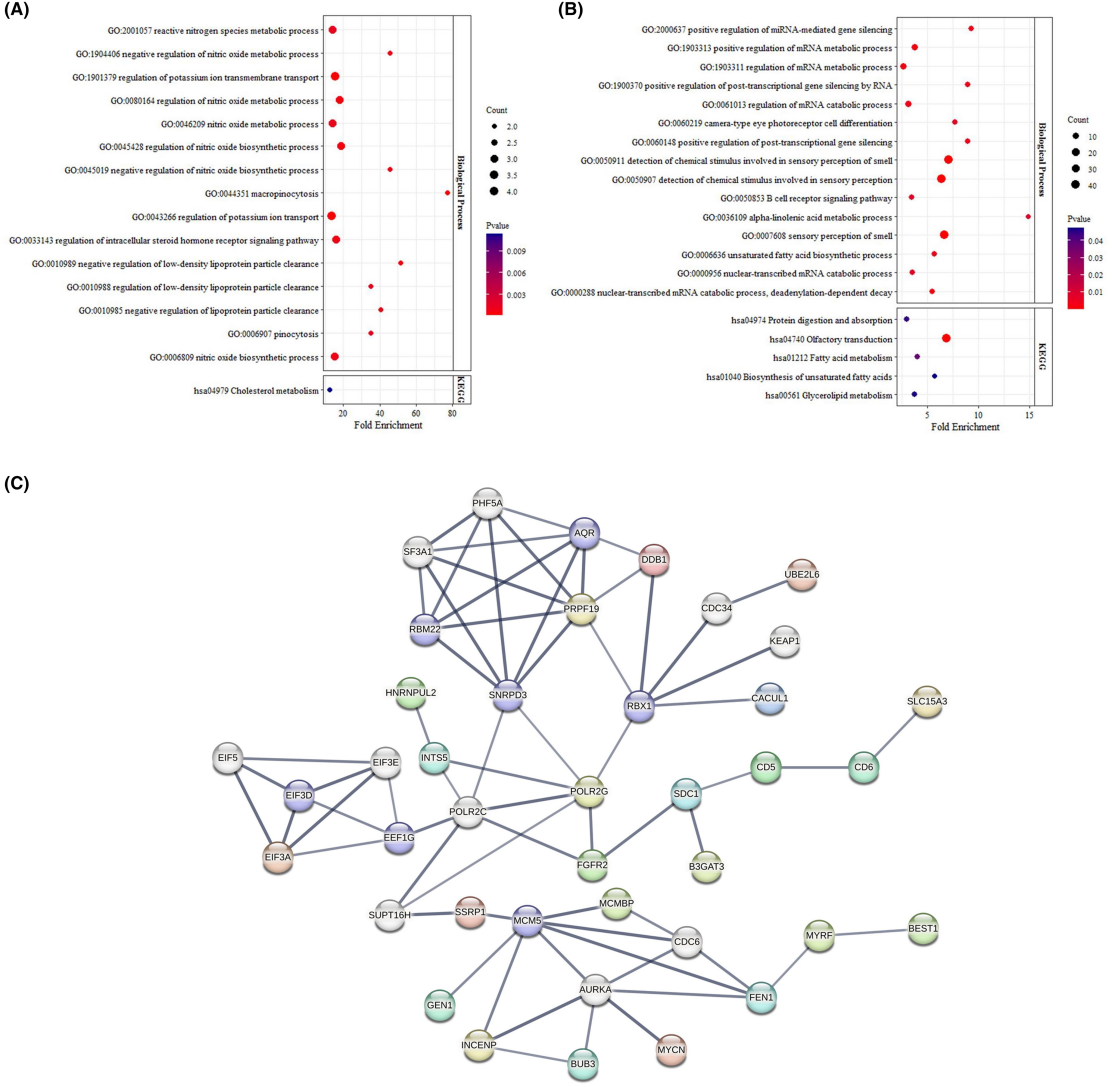
GO, KEGG and PPI analyses of ecDNA unique genes. (A) Biological process of GO annotation (Top 15) and KEGG pathway analysis (*p* < 0.05) of CON. (B) Biological process of GO annotation (Top 15) and KEGG pathway analysis (*p* < 0.05) of the LAA stroke patients. (C) PPI of LAA stroke ecDNA unique genes.

Considering the specific role of ecDNA (eccDNA size >100 kb), we further studied the ecDNA unique genes. Based on the results of GO annotation and KEGG pathway analysis, the LAA stroke ecDNA unique genes were significantly related to post‐transcriptional gene silencing, mRNA metabolic process, B‐cell receptor signalling, fatty acid/glycerolipid metabolism and biosynthesis of unsaturated fatty acid (Figure 5A,B), while the CON ecDNA unique genes were mainly involved in nitric oxide metabolic process, potassium ion transport, low‐density lipoprotein particle clearance and cholesterol metabolism. The unique genes of ecDNAs enriched in each process were shown in Table 2. And, based on the protein–protein interaction (PPI), we found several hubs of ecDNA unique genes in the LAA stroke patients, including SNRPD3, AQR, PRPF19, RBX1, POLR2C, POLR2G, EEF1G, MCM5 and AURKA (Figure 5C).

**TABLE 2:** Unique genes of ecDNAs in the LAA stroke patients and CON.

Group	Term	Gene
LAA	Post‐transcriptional gene silencing	MYCN/PUM2/TIAL1
	mRNA metabolic process	POLR2G/TNKS1BP1/PRPF19/CPSF7/SCGB1A1/PATL1/RC3H2/NBAS/PUM2/HMX2/NANOS1
	B‐cell receptor signalling	LPXN/MS4A1/IGHG4/IGHA1/IGHG2/PLEKHA1
	Fatty acid metabolism	FADS1/FADS3/ACADSB
	Biosynthesis of unsaturated fatty acid	FADS1/FADS3/FADS2/PTGS1
	Glycerolipid metabolism	LPIN1/PLPP4/TKFC
CON	Regulation of nitric oxide	MIR199A1/ACP5/DNM2
	Low‐density lipoprotein particle clearance	MIR199A1/LDLR
	Cholesterol metabolism	ANGPTL8/LDLR
	Potassium ion transport	DNM2/MIR103A1/KCNMB1/KCNIP1

## 4 DISCUSSION

EccDNAs, widely distributed in human tissues and cancerous tissues, might be biomarkers and therapy targets of ischemic stroke (IS), but its role in IS remains unknown. Thus, we firstly investigated the characteristics and potential function of plasma eccDNAs in patients with LAA stroke.

Compared with the healthy controls (CON), the size distribution and genomic characteristics of plasma eccDNA were not significantly altered in the LAA stroke patients. Similar with previous researches on plasma and cells/tissues from patients and healthy controls,^17^, ^18^, ^19^ the formation of eccDNAs was positively related to the distribution of genes on the chromosomes both in the CON and LAA stroke patients. The size of more than 90% eccDNAs was <1 kb, and two peaks were observed within 1 kb in the CON and LAA stroke patients. These data indicated that the formation of plasma eccDNAs was relatively stable during LAA stroke.

As DNA sequence providing binding sites for transcription factors, motif is a crucial feature of eccDNA. Transcription factors regulate gene expression by recognizing motifs located at regulatory regions and downstream chromatin processes. In the present study, two LAA stroke unique transcription factors, SHN3 and BCL6, were identified.

SHN3, a Schnurri family member of large zinc finger proteins, is potent to exert anti‐inflammation through blocking the functions of NF‐κB^20^ and increasing IL‐2 production of T cells.^21^ Several researches showed that inhibition of NF‐κB is an effective approach to reduce impairment of patients with either atherosclerosis^22^ or IS.^23^, ^24^ Clinical studies showed that the level of serum IL‐2 in patients with acute cerebral apoplexy was significantly decreased,^25^ and IS patients with poor outcomes had lower IL‐2 levels than those with good outcomes.^26^ Moreover, it has been reported that SHN3 accentuates transforming growth factor β (TGF‐β) signalling in epithelial cells,^27^ and activation of TGF‐β signalling pathway is significantly involved in the development of atherosclerosis^28^ and IS.^29^

BCL6, B‐cell lymphoma 6, is dysregulated after the onset of IS,^30^ and the effect of BCL6 in the IS is still controversial. Wei et al. reported that down‐regulation of BCL6 significantly reduced the size of cerebral infarction and oxidative stress levels in the brain of IS mice,^31^ while Zhou et al. found that up‐regulation of BCL6 inhibited inflammation and alleviated deleterious outcomes of IS.^32^ These data indicated that SHN3 and BCL6 could play a role in the pathogenesis of LAA and be a potential target to therapy for LAA stroke.

Based on the data of GO annotation and KEGG pathway analysis, the genes of differentially expressed eccDNAs between the LAA stroke patients and CON were involved in various progresses and signalling pathways related to IS, including focal adhesion, adherens junction, gap junction, Wnt, Rap1, ErbB, MAPK and calcium signalling. Of the molecules associated with focal adhesion, down‐regulation of talin and vinculin contributed to the instability of atherosclerotic plaques and increased risk of IS,^33^ and regulation of focal adhesion kinase manifested neuroprotective effect after IS.^34^ The molecules related to the adherens junction and gap junction, like catenin, are dysregulated after IS^35^ and could be promising targets for therapy. In addition, the effect of catenin in the IS was dependent on the Wnt signalling pathway,^36^ which was enriched by KEGG analysis of differentially expressed eccDNA genes. Furthermore, there also were several researches reporting that the Rap1, MAPK and calcium signalling were involved in the pathogenesis and therapeutic targets of IS.^37^, ^38^, ^39^ The results suggested that the levels of plasma eccDNAs were significantly associated with LAA stroke and potential to be used as biomarkers.

To further investigate the specific eccDNAs for LAA stroke, the unique genes were analysed in the LAA stroke patients and CON. Based on the GO and KEGG pathway analyses, the LAA stroke unique genes were enriched in regulation of haemostasis/coagulation and fibrinolysis, complement activation and lectin pathway, and IL‐10 production. Among the LAA stroke unique genes related to regulation of coagulation and fibrinolysis in the present study, CAV‐1 and vitronectin (VTN) could be potent therapy targets for IS.^40^, ^41^ Clarke et al. reported that, during periods of thrombolysis in IS, blocking complement cascade has been found to rescue tissue and improves functional outcome.^42^ In addition, the level of IL‐10 was significantly elevated after stroke,^43^ and low IL‐10 levels were related to poor outcomes of stroke.^44^

Due to the large size (>100 kb), ecDNAs could be the vehicles for genes (like oncogene and drug‐resistance genes in tumours) and enable them to be rapidly amplified, and lead to overexpression consequently. We also studied the role of ecDNAs in LAA stroke patients and found the LAA stroke ecDNA unique genes were significantly related to B‐cell receptor signalling, fatty acid/glycerolipid metabolism and biosynthesis of unsaturated fatty acid. Researches have been shown that B‐cell is involved with multiple stages of IS, including stroke risk factors, post‐stroke injury and repair.^45^ Hu et al. found that immunoglobulin heavy constant alpha 1 (IGHA1) related to B‐cell receptor signalling was upregulated in patients with IS and downregulated after rehabilitation,^46^ which may be contributed by the expression of IGHA1 carried by ecDNA in the LAA stroke patients. In addition, several researches reported that omega‐3 polyunsaturated fatty acids, like α‐linolenic acid, played a role in the prevention of stroke and manifested neuroprotective effect post‐stroke.^47^

Among the LAA stroke ecDNA unique genes, there were two hub genes significantly associated with atherosclerosis, including RNA polymerase II subunit C (POLR2C) and Aurora kinase A (AURKA). Ding et al. found that the expressions of POLR2C in plaques and serum from coronary atherosclerosis patients were significantly increased.^48^ Up‐regulation of AURKA resulted in dysregulated functions of endothelial cells and vascular smooth muscle cells, and promoted progression of atherosclerosis.^49^, ^50^ These ecDNAs carrying LAA stroke unique genes could be, by affecting the development of IS and atherosclerosis, involved with LAA stroke.

Besides LAA stroke ecDNA unique genes, the CON ecDNA unique genes were mainly involved in NO metabolic process and low‐density lipoprotein (LDL) particle. Declined level of NO contributing to endothelial dysfunction is a risk factor for IS and atherosclerosis,^51^, ^52^ which could be attributed by the absence of ecDNA‐carried genes related to the regulation of NO in the LAA stroke patients. Apart from NO, accumulation of oxidized LDL (oxLDL) are involved with the initiation and thrombosis of atherosclerosis,^53^ and higher combined serum oxLDL and LDL levels are associated with increased risk of recurrent stroke and poor functional outcomes in minor stroke.^54^ In IS mouse model, low‐density lipoprotein receptor (LDLR) was downregulated, and LDLR deficiency led to aggravated neurological deficits and long‐term cognitive dysfunction.^55^ In addition, the reduction of LDLR post IS may be resulted from the absence of LDLR‐contained ecDNAs in the LAA stroke patients. Therefore, aberrant NO and LDL metabolism could be, at least partially, contributed by the dysregulated ecDNAs in the development of LAA stroke, and the ecDNAs could be potentially therapeutic molecules in LAA stroke.

Lastly, based on the LAA stroke or CON unique genes, five LAA stroke unique eccDNAs (Chr2:12724406‐12724784, Chr4:1867120‐186272046, Chr4:186271494‐186271696, Chr7:116560296‐116560685 and Chr11:57611780‐5761192) and three CON unique eccDNAs (Chr10:133363251‐133363389, Chr16:67405842‐67406042 and ChrX:129832187‐129832537) were identified in the present study. Although, due to heterogeneity of eccDNA among diseases, these unique eccDNAs were not matched in two public eccDNA database, eccDNAdb and CircleBase, they could be prognosis biomarkers and therapeutic targets for LAA stroke once verified in more LAA stroke patients.

There are two limitations in the present study. Firstly, five unique eccDNAs of LAA stroke were based on five patients and five controls, and more samples are needed to evaluate and validate the data in the future. Secondly, the mechanisms of differentially expressed eccDNAs between LAA stroke patients and controls were not validated by experiments in vivo or in vitro, which would be the focus of future research.

## 5 CONCLUSION

Our data firstly revealed the characteristics and specific expression profiles of plasma eccDNA in LAA stroke patients, enabling us to explore the potential biomarkers and therapeutic targets of LAA stroke unique eccDNAs and eccDNA genes in the pathogenesis of LAA stroke. Further study is worthy to analyse and prove the significance of eccDNA in the regulatory mechanisms of LAA stroke.

## AUTHOR CONTRIBUTIONS

**Kejie Chen:** Conceptualization (supporting); formal analysis (lead); methodology (equal); visualization (equal); writing – original draft (equal); writing – review and editing (equal). **Yanqi Chi:** Formal analysis (supporting); validation (equal); visualization (equal); writing – original draft (equal). **Hang Cheng:** Formal analysis (supporting); validation (equal); visualization (equal); writing – original draft (equal). **Min Yang:** Validation (supporting). **Quandan Tan:** Validation (supporting). **Junli Hao:** Validation (supporting). **Yapeng Lin:** Validation (supporting). **Fengkai Mao:** Validation (supporting). **Song He:** Validation (supporting). **Jie Yang:** Conceptualization (lead); formal analysis (equal); funding acquisition (equal); methodology (equal); writing – original draft (equal); writing – review and editing (equal).

## FUNDING INFORMATION

This research was funded by the National Natural Science Foundation of China, grant number 82171295; Sichuan Science and Technology Program, grant numbers 2023YFS0042 and 2023NSFSC1462.

## CONFLICT OF INTEREST STATEMENT

The authors confirm that there are no conflicts of interest.

## PATIENT CONSENT STATEMENT

Written informed consent has been obtained from the patients to publish this paper.

## Supporting information


**FIGURE S1.** Distribution of plasma eccDNAs on chromosomes. (A) Distribution of eccDNAs from the LAA stroke patients and CON on each chromosome. (B) Numbers of eccDNAs per Mb on each chromosome of LAA stroke patients and CON. (C) Percentages of coding gene per Mb on each chromosome of LAA stroke patients and CON.
**FIGURE S2.** Size distribution of plasma eccDNAs. (A) Percentages of different size of eccDNAs from LAA stroke patients and CON. (B) Length of eccDNA from different size of eccDNAs. (C–F) Density plots of eccDNA size of LAA stroke patients and CON.

## Data Availability

The data that support the findings of this study are available from the corresponding author upon reasonable request.
