# Supplementary material for: Identification and characterization of extrachromosomal circular DNA in large‐artery atherosclerotic stroke
Source: J Cell Mol Med. 2024 Mar 20;28(7):e18210. doi: 10.1111/jcmm.18210 (PMC10951879; doi:10.1111/jcmm.18210)
Supplement: Supplementary file 1 — FIGURE S1. Distribution of plasma eccDNAs on chromosomes. (A) Distribution of eccDNAs from the LAA stroke patients and CON on each chromosome. (B) Numbers of eccDNAs per Mb on each chromosome of LAA stroke patients and CON. (C) Percentages of coding gene per Mb on each chromosome of LAA stroke patients and CON. FIGURE S2. Size distribution of plasma eccDNAs. (A) Percentages of different size of eccDNAs from LAA stroke patients and CON. (B) Length of eccDNA from different size of eccDNAs. (C–F) Density plots of eccDNA size of LAA stroke patients and CON. [file JCMM-28-e18210-s001.docx]

**Appendices**


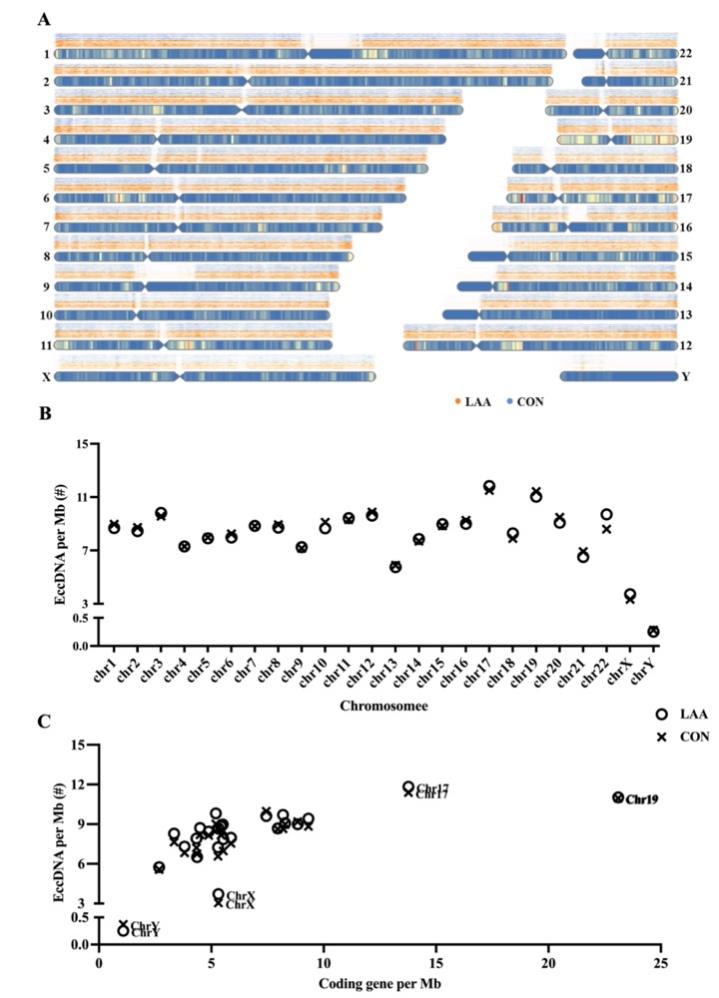


Supplementary Figure 1. Distribution of plasma eccDNAs on chromosomes. (A) Distribution of eccDNAs from the LAA stroke patients and CON on each chromosome. (B) Numbers of eccDNAs per Mb on each chromosome of LAA stroke patients and CON. (C) Percentages of coding gene per Mb on each chromosome of LAA stroke patients and CON.


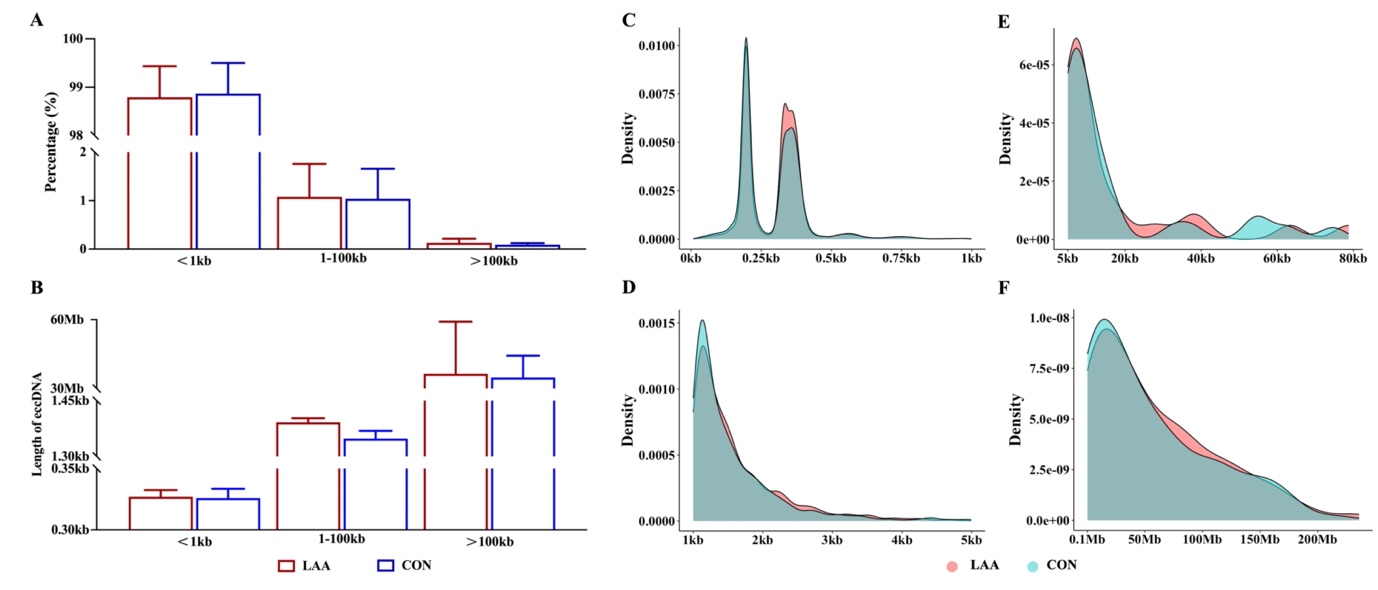


Supplementary Figure 2. Size distribution of plasma eccDNAs. (A) Percentages of different size of eccDNAs from LAA stroke patients and CON. (B) Length of eccDNA from different size of eccDNAs. (C-F) Density plots of eccDNA size of LAA stroke patients and CON.
